# Data for analysis of catechol estrogen metabolites in human plasma by liquid chromatography tandem mass spectrometry

**DOI:** 10.1016/j.dib.2019.103740

**Published:** 2019-03-08

**Authors:** Nina Denver, Shazia Khan, Ioannis Stasinopoulos, Colin Church, Natalie Z.M. Homer, Margaret R. MacLean, Ruth Andrew

**Affiliations:** a Mass Spectrometry Core, Edinburgh Clinical Research Facility, Queen's Medical Research Institute, 47 Little France Crescent, Edinburgh, EH16 4TJ, United Kingdom; b Institute of Cardiovascular and Medical Sciences, College of Medical, Veterinary and Life Sciences, University of Glasgow, University Avenue, Glasgow, G12 8QQ, United Kingdom; c Strathclyde Institute of Pharmacy and Biomedical Sciences, University of Strathclyde, 161 Cathedral Street, Glasgow, G4 0RE, United Kingdom; d Scottish Pulmonary Vascular Unit, Golden Jubilee National Hospital, Agamemnon St, Clydebank, G81 4DY, United Kingdom

## Abstract

Analysis of catechol estrogens (2 & 4 hydroxy-estrone and estradiol) has proven troublesome by liquid chromatography tandem mass spectrometry due to their low concentrations, short half-lives and temperature-labile nature. Derivatization to methyl piperazine analogues has been reported for a panel of 9 estrogens in, “Derivatization enhances analysis of estrogens and their bioactive metabolites in human plasma by liquid chromatography tandem mass spectrometry” (Denver et al., 2019). Data show alteration of the base catalyst in this method was required to allow detection of catechol estrogens to low levels. Data also highlight the challenges faced in chromatographic separation of isomers and isotopologues, which were partially overcome by employing an extended column length and reduced oven temperature. In addition, data analysis displayed significant matrix effects during quantitation in plasma, following solid-phase extraction, despite efficient recoveries.

**Table:** Specifications table

Subject area	*Chemistry*
More specific subject area	*Analytical, Bioanalytical and Clinical Chemistry*
Type of data	*Tables, figure*
How data was acquired	*Liquid Chromatography, Mass Spectrometry (LC-MS/MS)*
Data format	*Analyzed Data*
Experimental factors	*Experiments for extraction of catechol estrogens from plasma for LC-MS/MS analysis*
Experimental features	*Development of quantitative approach for catechol estrogens (2OHE1, 4OHE1, 2OHE2 and 4OHE2).*
Data source location	*Scottish Pulmonary Vascular unit, Golden Jubilee National Hospital, Agamemnon St, Clydebank, Glasgow, G814DY*
Data accessibility	*Data in the article*
Related research article	N. Denver, S. Khan, I. Stasinopoulos, C. Church, N.Z. Homer, M.R. MacLean, R. Andrew, Derivatization enhances analysis of estrogens and their bioactive metabolites in human plasma by liquid chromatography tandem mass spectrometry, Anal. Chim. Acta. 1054 (2019) 84–94.

**Table:** 

**Value of the data** • Illustrates a common problem faced in quantitative estrogen metabolite assays for catechol estrogens • LC-MS/MS parameters are reported for identification and resolution of catechol metabolites • The derivatization method allows analyte detection to 20 pg mL^−1^ in aqueous solutions • Recovery and ion suppression data for researchers considering solid phase extraction of these analytes

## 1 Data

**Fig. 1:**
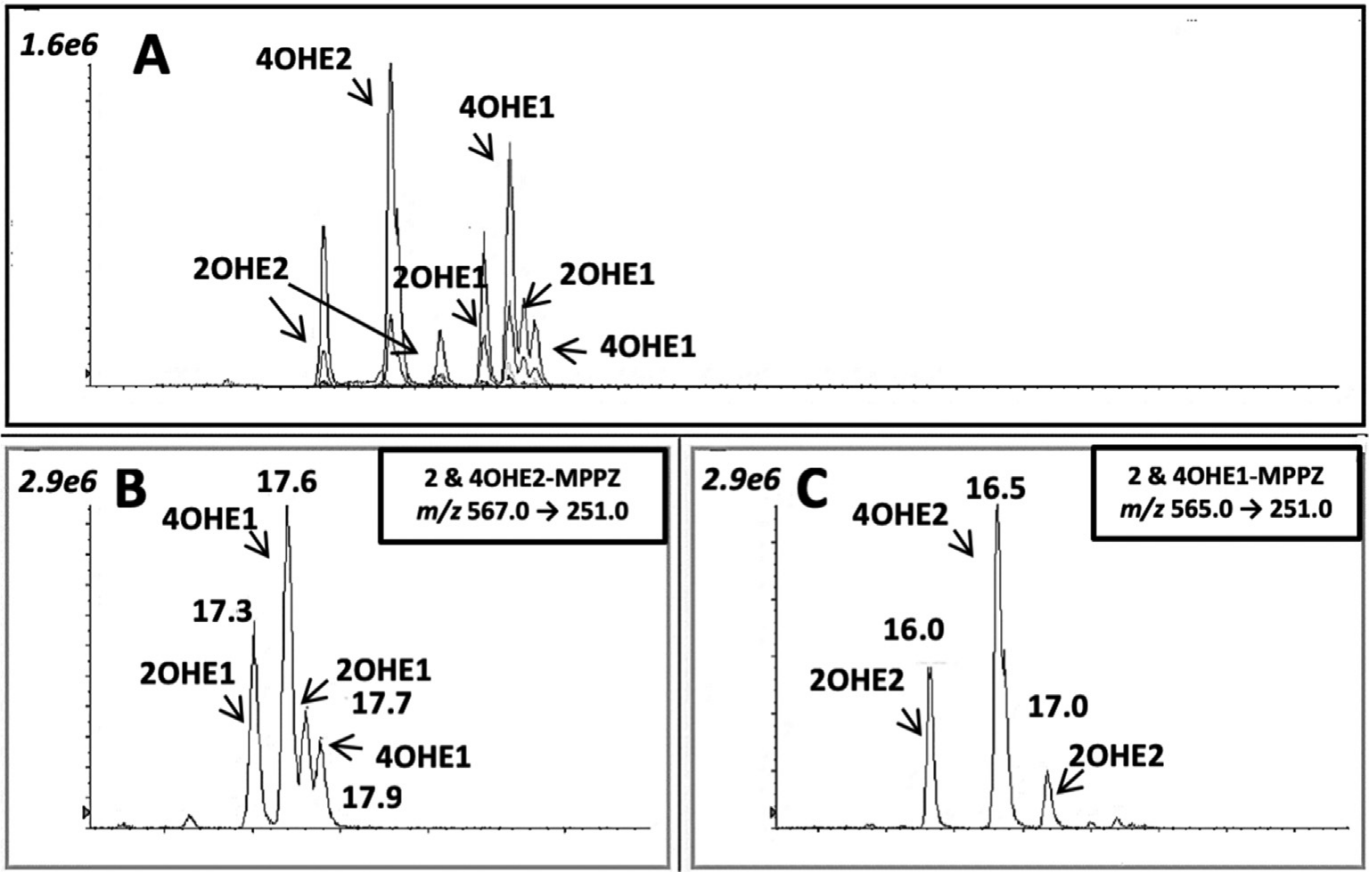
Total and extracted ion chromatograms of (A) methylpiperazine (MPPZ) derivatives of catechol estrogens, (B) The estrone metabolites 2-Hydroxyestrone (2OHE1), 4-Hydroxyestrone (4OHE1), and (C) the estradiol metabolites 2-Hydroxyestradiol (2OHE2), 4-Hydroxyestradiol (4OHE2) at 1000 pg mL^−1^. Figure illustrating challenges in separating catechol metabolites by mass transitions and retention time (min).

Here we display data in Table 1, which illustrates the mass spectrometry tuning parameters of MPPZ-derivatives of catechol estrogens, shown by their exact theoretical and observed masses. In Table 2, data demonstrating the limit of detection that can be achieved for analytes following derivatization are given for unextracted standards, alongside observed retention times from chromatographic interpretation, Fig. 1. Finally, the recoveries of catechol estrogens from plasma following solid-phase extraction (SPE) are displayed in Table 2, with associated data describing matrix effects.

**Table 1:** Mass spectrometric analysis of MPPZ derivatized catechol estrogens.

Analyte-MPPZ	Accurate mass precursor ion *m*/*z*	Theoretical product ion mass	Observed product ion mass *m*/*z*	Product ion Δ ppm	Collision energy (V)	Collision exit cell potential (V)	De-clustering potential (V)
2OHE1	565.2662	^a^251.1269 ^b^58.0656	^a^251.1276 ^b^58.0651	2.78 8.61	59.0 129.0	10.0 10.0	130.0 130.0
4OHE1	565.2662	^a^251.1269 ^b^58.0656	^a^251.1274 ^b^58.0673	0.39 29.27^c^	59.0 129.0	10.0 10.0	130.0 130.0
2OHE2	567.2819	^a^251.1269 ^b^281.1249	^a^251.1274 ^b^281.1252	1.99 1.06	61.0 61.0	22.0 22.0	166.0 166.0
4OHE2	567.2819	^a^251.1269 ^b^281.1249	^a^251.1265 ^b^281.1251	1.59 0.71	61.0 61.0	22.0 22.0	166.0 166.0
^13^C_6-_4OHE1	571.2864	^a^251.1269 ^b^58.0656	^a^251.1268 ^b^58.0661	0.39 1.72	50.0 100.0	15.0 15.0	136.0 136.0
^13^C_6-_2OHE2	573.3020	^a^251.1269 ^b^281.1249	^a^251.1269 ^b^281.1251	0.00 0.71	50.0 50.0	15.0 15.0	136.0 136.0

^a^quantifier ion, ^b^qualifier ion, ^c^Fragments with low signal intensity following infusion generated higher ppm values; Entrance potential = 10V; mass to charge (*m*/*z*); Mass error (Δppm); Voltage (V); 2-hydroxyestrone (2OHE1); 4-hydroxyestrone (4OHE1); 2-hydroxyestradiol (2OHE2); 4-hydroxyestradiol (4OHE2); 13,14,15,16,17,18-^13^C_6_-4-hydroxyestrone (^13^C_6_-4OHE1); 13,14,15,16,17,18-^13^C_6_-2-hydroxyestradiol (^13^C_6_-2OHE2); MPPZ, 1-(2, 4-dinitrophenyl)-4,4-dimethylpiperazine.

**Table 2:** Indices of extraction performance.

Analyte-MPPZ	Internal standard	Unextracted LOD (pg mL^−1^)	Retention time (s) (min)	MCX^®^ recovery (%)	Generic IonSup (%)	Optimized IonSup (%)
2OHE1	^13^C_6_-4OHE1	20	17.38/17.75	72 ± 3	−94 ± 6	−72 ± 2
4OHE1	^13^C_6_-4OHE1	20	17.62/17.90	68 ± 2	−94 ± 2	−73 ± 4
2OHE2	^13^C_6_-2OHE2	20	16.03/17.00	69 ± 2	−93 ± 8	−69 ± 8
4OHE2	^13^C_6_-2OHE2	20	16.58	62 ± 4	−95 ± 9	−71 ± 6

2-hydroxyestrone (2 OHE1); 4-hydroxyestrone (4 OHE1); 2-hydroxyestradiol (2 OHE2); 4-hydroxyestradiol (4 OHE2); 13,14,15,16,17,18-^13^C_6-_4-hydroxyestrone (^13^C_6_-4OHE1); 13,14,15,16,17,18-^13^C_6_-2-hydroxyestradiol (^13^C_6_-2OHE2); MPPZ, 1-(2, 4-dinitrophenyl)-4,4-dimethylpiperazine; LOD, Limit of detection; min, minutes; MCX, Mixed Cation Exchange; IonSup, Ion Suppression.

Catechol estrogens (2 and 4-hydroxy-estrogens) are challenging metabolites to analyze by LC-MS/MS [2], [3]. Common analytical challenges arise due to their unstable nature and short half-lives [4]. Derivatization to 1-(2, 4-dinitrophenyl)-4,4-dimethylpiperazinium (MPPZ) derivatives has been successfully applied for analysis of estrone, estradiol, 16-hydroxy and 2 and 4 methoxyestrogens [1]. Here the successes and pitfalls of applying this approach to analyse catechol estrogens are described. For 2 &4-hydroxyestrogens, MPPZ derivatives were generated with the original protocol [1] but with poor yield, with insufficient detection upon lowering the concentrations (<500 pg mL^−1^). Comparison of various derivatization base catalysts (sodium bicarbonate, triethylamine, pyridine, ammonium hydroxide and *N*-diethylaniline) was key in achieving efficient derivatization. Modification of the base catalyst to *N*-diethylaniline enhanced PPZ derivatization with catechol estrogens, showing ×500 increase in peak area response, but this approach caused reduction of signals of the 9 other estrogens (E1, E2, 16-hydroxy and 4 and 2-methoxy-estrogens) within this sex steroid pathway. Combinations of base catalysts were also tested to create a holistic approach. However, derivatization of the catechol metabolites and additional 9 estrogens were only successful in separate reactions.

Structural identification of precursor and product ions for the catechol derivates were achieved by high resolution MS and multiple reaction monitoring for quantitation established by triple quadrupole MS, Table 1.

Double derivatives of these compounds were not seen, but isomeric mono-derivatives were observed, believed due to the possibility of either of the A-ring hydroxyl groups reacting. To achieve the highest degree of chromatographic resolution of isomers and isotopologues, a C18_PFP (2.1 × 150mm) was coupled to a C18_PFP (2.1 × 20mm). The reduction of oven temperature from 25 to 20 °C also aided in resolving the catechol estrogen derivative peaks (Fig. 1).

Data illustrated that recovery of catechol estrogens (pre vs post-spiked PA) from Oasis MCX cartridges, Table 2 was acceptable (2OHE1 73%, 4OHE1 66%, 2OHE2 68% & 4OHE 64%). However, unfortunately significant ion suppression (unextracted peak area vs extracted + derivatized estrogen peak area) was present for all catechol metabolites recovered from plasma (2OHE1 by 94%, 4OHE1 96.1%, 2OHE2 94.7% & 4OHE2 96.5%), Table 2. Additional clean up steps utilizing aqueous methanol or acetonitrile (0–70% v/v) improved ion suppression but not to an acceptable degree; the least ion suppression was observed with washes of 60%v/v MeOH (Ion suppression: 2OHE1 by 80.5%, 4OHE1 78.9%, 2OHE2 79.9% & 4OHE2 79.7%) and 30% ACN (2OHE1 by 80%, 4OHE1 88%, 2OHE2 77% & 4OHE2 86%). Further modification of elution solvent (70–100% v/v MeOH) did not decrease ion suppression sufficiently with the optimal wash of 95% v/v MeOH still showing suppression of ∼80% (2OHE1 85%, 4OHE1 95%, 2OHE2 78% & 4OHE2 71%). Thus, an alternative extraction protocol for use alongside the modified MPPZ derivatization protocol for 2, 4 hydroxy estrogens is required.

## 2 Experimental design, materials, and methods

### 2.1 Materials

2-Hydroxyestrone (2OHE1), 4-hydroxyestrone (4OHE1), 2-hydroxyestradiol (2OHE2), 4-hydroxyestradiol (4OHE2) were from Steraloids, Inc (Newport, USA). 13,14,15,16,17,18-^13^C_6_-4-Hydroxyestrone (^13^C_6_-4OHE1) and 13,14,15,16,17,18-^13^C_6_-2-hydroxyestradiol (^13^C_6_-2OHE2) were from CK Isotopes Limited (Leicestershire, UK). *N*-Diethylaniline was from Acros Organics (Geel, Belgium). All additional reagents were sourced as specified in Denver et al. [1].

### 2.2 Methods

Analysis, including assessment of extraction efficiency and ion suppression, was performed according to the approach described in Denver et al. [1] and modifications for catechol estrogens reported below.

#### 2.2.1 Instrumentation

Structures of fragment ions formed from estrogen derivatives were determined by high resolution MS using a SYNAPT G2Si instrument (Waters Corp, Manchester, UK) fitted with an ESI source in positive mode [1]. Method development was performed using a Shimadzu Nexera X2 LC (Shimadzu, Kyoto, Japan) coupled to a Sciex 6500 + Mass Spectrometer (Sciex, Warrington, UK) operated in positive electrospray (ESI).

#### 2.2.2 Chromatographic conditions

Estrogen metabolites were analyzed both individually and in a mixed solution to confirm separation. Two Ace Excel 2 C18-PFP column (150 × 2.1 mm, 2 μm + 20 × 2.1 mm, 2 μm; HiChrom, Reading, England) were coupled at an oven temperature of 20 °C. A gradient solvent system of water: acetonitrile (90:10), containing formic acid (FA; 0.1%, 0.5 mL/min) was diverted to waste for the initial 9 minutes followed by elution for a further 4 minutes at 90:10, then with a gradient over 3 minutes until final conditions of water: acetonitrile (90:10) containing FA (0.1%, 0.5 mL/min) were achieved. Injection volume was 30 μL.

#### 2.2.3 Derivatization and optimization

The following protocol was applied for derivatization PPZ stock (10 μL; 1mg mL^−1^), *N*-diethylaniline (10 μL) and acetone (70 μL) were added to the catechol estrogen standards and was capped and incubated (60 °C, 1 h). Reagents were reduced to dryness at 40 °C under oxygen free nitrogen (OFN). The dried residue was incubated (40 °C, 2 h) with CH_3_I (100 μL). The mixture was reduced to dryness under OFN and dissolved in H_2_O/CH_3_CN (70:30; 70 μL).

#### 2.2.4 Extraction and optimization

SPE using Oasis^®^ MCX (3 cc/60 mg, Waters, Wilmslow, UK) cartridges was applied under gravity. Prior to loading the sample, cartridges were conditioned and equilibrated with methanol (2 mL), followed by water (2 mL). The diluted sample (0.5 mL plasma + 0.5mL water (or 1 mL water for standards) + 200 pg mL^−1^ Internal Standard) was loaded and allowed to pass through the cartridges and the eluate discarded. The cartridges were washed with aqueous FA (2% v/v, 2 mL). A second wash of methanol (60% v/v, 2 mL) was applied with the eluate discarded. Steroids were eluted in methanol (95%; 2 mL). Extracts were reduced to dryness under OFN (40 °C) and the residues were derivatized as above.
